# Clear Aligner Treatment: Indications, Advantages, and Adverse Effects—A Systematic Review

**DOI:** 10.3390/dj13010040

**Published:** 2025-01-17

**Authors:** Clara Rasborg Hartogsohn, Liselotte Sonnesen

**Affiliations:** Section of Orthodontics, Department of Odontology, Faculty of Health and Medical Sciences, University of Copenhagen, DK-2200 Copenhagen, Denmark; clara.hartogsohn@sund.ku.dk

**Keywords:** orthodontics, clear aligner treatment, malocclusions

## Abstract

**Background/Objectives**: Clear aligner treatment (CAT) has gained interest among clinicians as well as among patients. The aim of the present study was to systematically review the literature regarding current viewpoints on indications, contraindications, advantages, disadvantages, and adverse effects in CAT. **Methods**: A search was performed in the PubMed and Embase databases, yielding 18 studies eligible for inclusion. **Results**: Current indications for CAT are mild to moderate malocclusions. Severe malocclusions with impactions or severe craniofacial skeletal deviations are contraindications. The advantages were oral hygiene and oral health as the most common, and disadvantages of CAT have been noted by several studies, including CAT still being inferior to fixed appliance treatment (FAT) in all orthodontic movements. Adverse effects were potential health risks due to microplastics and a decrease in condyle bone volume. **Conclusions**: No firm conclusions can be drawn regarding indications for CAT except for mild to moderate malocclusion. Severe malocclusions with impactions or severe craniofacial skeletal deviations are considered contraindications. In more complex cases, CAT is still considered inferior to FAT, although CAT contains advantages. There are disadvantages in CAT which clinicians should consider when choosing a type of orthodontic appliance for treating specific malocclusions. Only a few adverse effects in CAT were presented by the included studies. More high-quality research is needed regarding indications and contraindications for CAT.

## 1. Introduction

The popularity of orthodontic treatment with clear aligners has been increasing among patients as well as among clinicians. Since the introduction of Kesling’s “tooth positioning appliance” in 1945, the idea of moving teeth using a series of trays instead of fixed orthodontic appliances has been under development and improved upon ever since [1]. In 1997, Invisalign^®^ was introduced to the market along with the idea of the clear aligner treatment (CAT) we know today [1]. The reason behind the increasing interest in CAT might be explained by the more esthetic appearance and low visibility of the appliance compared to conventional fixed orthodontic appliances. Appearance is of high value in our contemporary world, not least due to the impact of social media with its high focus on looks and image [2].

In orthodontic treatment, understanding and respecting biological and biomechanical principles are greatly important, which, naturally, also applies to CAT. Thus, diagnostics and treatment planning are essential in achieving acceptable and successful results [3]. In recent studies [4,5,6,7], it has been suggested that patients receiving CAT are experiencing fewer inconveniences, less pain, fewer problems with eating and speaking, and a better oral-health-related quality of life (OHRQoL). In general, there are studies [4,7,8] indicating that patients receiving CAT experience a more comfortable treatment compared to patients receiving fixed appliance treatment (FAT). However, studies have also found minimal or no differences between the two treatment modalities [6,9]. CAT is still considered to be relatively new in orthodontics, and its effectiveness, limitations, advantages, disadvantages, and adverse effects are not yet fully known or sufficiently documented in the literature [1].

The aim of the present study was to systematically review the literature to determine the current aspects of CAT, including indications/contraindications, advantages/disadvantages, and adverse effects.

## 2. Materials and Methods

This systematic review is reported according to the Preferred Reporting Items for Systematic Reviews and Meta-Analyses (PRISMA) guidelines [10]. This review is following the PRISMA guidelines. No review protocol was prepared.

### 2.1. Inclusion and Exclusion Criteria

The inclusion and exclusion criteria were as follows:

Inclusion:

‑ Original studies investigating indications, contraindications, advantages, disadvantages, and/or adverse effects in CAT;
‑ Original studies published in 2016 or later;
‑ Original studies including a control group;
‑ Publications available in English.

Exclusion:

‑ Systematic reviews and meta-analyses;
‑ Case reports and case series;
‑ Original studies published before 2016;
‑ Original studies with no control group;
‑ Original studies exclusively investigating primary or mixed dentition;
‑ Publications not available in English.

Furthermore, the Population, Intervention, Comparison, and Outcomes (PICO) framework was used to define the in- and exclusion criteria as well as to address the aims of this systematic review:

Population: Patients/individuals with permanent dentition;

Intervention: CAT;

Comparison: No treatment or conventional orthodontic treatment;

Outcomes: Indications, contraindications, advantages, disadvantages, and/or adverse effects in CAT.

### 2.2. Data Sources

A search was conducted in the PubMed and Embase databases with a date limit set to 2016. The date limit was set to 2016 since Invisalign^®^’s “Generation 8” (G8) represents the current generation of aligners on the market and the predecessor, G7, was marketed in 2016. The last search was conducted on the 14 of November 2024.

### 2.3. Search Strategies and Screening

**Figure 1:**
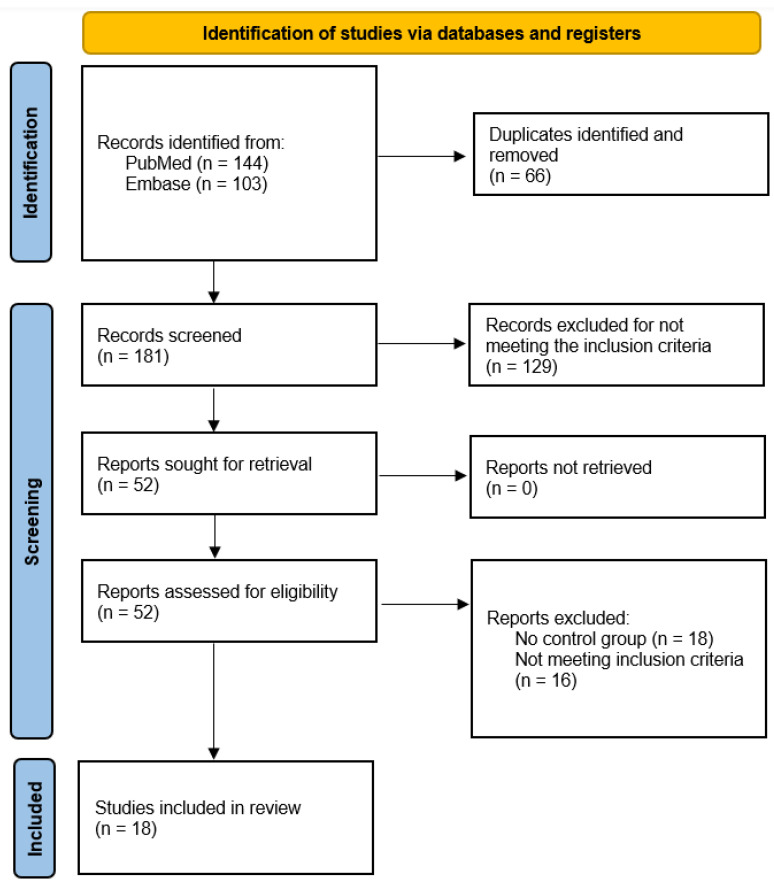
PRISMA 2020 flow diagram of the study selection process [11].

All terms related to CAT, indications, contraindications, advantages, disadvantages, and adverse effects were included as MeSH terms and/or Textwords/Keywords combined using ‘OR’ and ‘AND’ (Table 1). The studies were initially screened by title and abstract based on the in- and exclusion criteria. Studies initially meeting the inclusion criteria were included for full-text screening. Two reviewers (C.R.H and L.S.) screened the abstracts, and the studies were then retrieved for full-text screening. The screening procedure and study selection are displayed in a flow diagram (Figure 1).

### 2.4. Data Extraction

The data extracted from each included study consisted of first author, year of publication, type of study, study objectives and outcome related to indications, contraindications, advantages, disadvantages, and adverse effects in CAT (Table 2, Table 3, Table 4, Table 5 and Table 6). Both reviewers (CRH and LS) were responsible for the data extraction.

### 2.5. Quality Assessment

Four randomized clinical trials (RCTs) [16,22,23,26] were included in this review. Other studies were of lower quality compared to the RCTs, such as non-randomized controlled clinical trials and retrospective, prospective, and cross-sectional studies. Eight studies [12,14,17,18,19,22,24,27] had sample sizes >60 patients, whereas nine of the included studies [13,15,16,20,21,23,25,26,29] had sample sizes ranging from 28 to 41 patients. Both reviewers (CRH and LS) assessed the quality of the studies.

## 3. Results

A total of 247 studies were identified following the initial search in the PubMed and Embase databases. Title and abstract screening resulted in 52 studies included for full-text reading. Of the 52 studies, 18 studies were excluded due to a lack of control group and 16 studies were excluded for not meeting the other inclusion criteria. Eighteen original studies met all the inclusion criteria and were included for analysis. See Figure 1 for a flow diagram of the included studies.

### 3.1. Study Characteristics

The main findings and descriptive data of the included studies are summarized in Table 2, Table 3, Table 4, Table 5 and Table 6.

### 3.2. Results Regarding Indications for CAT

Among the studies included, indications for CAT were only highlighted in a single study [12] as mild to moderate malocclusions (Table 2).

### 3.3. Results Regarding Contraindications for CAT

Abu-Arqub et al.’s study (2023) [12] is the only one to mention that complex malocclusions with impactions or severe craniofacial skeletal deviations are considered contraindications for CAT (Table 3).

### 3.4. Results Regarding Advantages of CAT

Some of the recurrent advantages of CAT highlighted by three studies [13,16,22] are oral hygiene and oral health. These studies find that patients undergoing CAT have a better oral health status compared to patients undergoing FAT. Furthermore, the levels of oral cariogenic bacteria are lower in patients receiving CAT compared to patients receiving FAT [13]. One study [19] also finds that the risk of developing caries in general while undergoing orthodontic treatment was lower in CAT compared to FAT (Table 4).

Two studies [16,18] found that OHRQoL was better in patients undergoing CAT compared to the FAT groups, and a single study [17] found that clear aligners were more esthetically pleasing (Table 4).

Two studies [14,20] found that orthodontically induced external apical root resorption (OIEARR) was less severe and rarer compared to the level of OIEARR observed in FAT (Table 4). Nevertheless, OIEARR was still present in CAT, and one study [27] claimed that patients receiving CAT had a similar predisposition to OIEARR as patients receiving FAT (Table 5).

A significantly higher reduction in the Peer Assessment Rating (PAR) index from T0 to T1 in CAT vs. FAT was observed by a single study [15] (Table 4).

Less discomfort was also found as an advantage in one study [23]. In addition, this study found that patients in the CAT group had a lower need for pain medication during treatment compared to the FAT group.

A positive association between body posture, spine position, and occlusal contacts was found to be better in the Invisalign^®^ group compared to untreated patients [21] (Table 4). According to the authors, their study demonstrated that treatment with Invisalign^®^ resulted in a positive correspondence between these parameters (after treating class I malocclusion with crowding for six months). The authors also stated that “Our preliminary results seem to indicate that orthodontic appliances able to disclose the jaws, improving intercuspation can affect the biting efficiency during function, thus resulting in more controlled neuromuscular co-contractions patterns probably producing a more balanced posture” [21]. The difference between th study and the control group was significant after six months of treatment.

### 3.5. Results Regarding Disadvantages of CAT

One study [24] found higher scores in the Pittsburgh Sleep Quality Index (PSQI) and on the Epworth Sleepiness Scale (ESS) among the CAT group compared to the FAT group, although no significant differences were found (Table 5).

Changes in speech were found to be significantly greater in CAT by two studies [25,26] (Table 5).

FAT was found by most orthodontists to have better control for all tooth movements and resulted in better occlusal contacts than CAT [12]. It was also stated that several refinements are often required in CAT to reach the desired goal. Furthermore, it is challenging to perform root movements and extrusions with clear aligners [12] (Table 5).

As mentioned earlier, a single study [27] found that patients receiving CAT have the same predisposition to OIEARR as patients receiving FAT (Table 5).

### 3.6. Results Regarding Adverse Effects in CAT

One study [28] found that microparticles <5 µm from clear aligners can cross membranes and therefore pose a potential health risk in CAT since the ingestion of microplastics might cause oxidative stress and inflammation (Table 6). In addition, the human immune system is unable to remove synthetic particles, which could lead to chronic inflammation and increase the risk of neoplasia [28].

Another study [29] found that the mandibular condyle bone of patients being treated with clear aligners was reduced in volume compared to a group being treated with FAT (Table 6).

## 4. Discussion

### 4.1. Indications for CAT

According to one of the included studies [12] in the present systematic review, the indication for CAT is mild to moderate malocclusion. This is in accordance with the overview of systematic reviews by Yassir et al. (2022) [30] and the recommendations for CAT from the Angle Society of Europe [1], who recommend CAT for mild malocclusions, where they find that clear aligners perform just as well as FAT. The same is highlighted in the systematic review by Caruso et al. (2024) [31], who also states that the only clear indication for CAT currently is mild to moderate malocclusions in non-extraction and non-growing patients. Apart from that, no clear clinical recommendations can be made regarding CAT according to this study [31] if they are to be based on solid and high-quality scientific evidence.

### 4.2. Contraindications for CAT

A single study [12] emphasizes that complex malocclusions with impactions, extractions, and severe skeletal deviations are often avoided in relation to CAT by orthodontists. This is in accordance with the current literature [1,30], which also states that CAT should be used with caution in severe malocclusions, especially if they require extractions. CAT is still considered inferior to FAT in achieving torque control, optimal root approximation, and contact point relations [1,30]. However, in the study by Jaber et al. (2023) [32], it was concluded that CAT was just as effective as FAT in treating premolar extraction-based cases, though FAT was considered better in achieving buccolingual inclination and occlusal contacts in a shorter treatment duration. Furthermore, it was noted that a discrepancy between the expected outcome and the achieved outcome might be associated with CAT [32].

### 4.3. Advantages of CAT

An advantage of orthodontic treatment with clear aligners which was highlighted was the ability to maintain better oral hygiene and oral health compared to FAT [13,16,22]. Oral hygiene is easier to maintain when receiving CAT compared to FAT since the clear aligners are removable, which enables the patient to brush and clean approximately as usual. This is supported by the existing literature [30,33], which also states that CAT allows for better oral hygiene maintenance and therefore better periodontal health compared to FAT.

OHRQoL was stated by two studies [16,18] to be higher in CAT compared to FAT. Clear aligners are often considered to be a more esthetically pleasing appliance due to their “transparent” nature and have, in general, been advantageous regarding physical pain, psychological inconveniences, as well as in other aspects of physical, psychological, and social elements [34]. Other studies [4,30] also find that the OHRQoL scores are higher in CAT compared to FAT. On the contrary, one study [6] found no difference in the OHRQoL scores between a group treated with brackets and a group treated with Invisalign^®^, except for eating and chewing.

According to the included studies [14,20,27], OIEARR is present in CAT even though it might be less severe and less frequently observed when compared to FAT. These findings are confirmed by the existing literature [1,30]. The reason for this might be that less severe malocclusions are treated with CAT compared to the malocclusions treated with FAT and therefore require fewer complex movements.

A higher reduction in the PAR index from T0 to T1 was found by a single study [15]; however, at the end of the treatment, there was no significant difference between the PAR index in the two groups. Similar findings have been found in a recent study [35] using the PAR index to compare the outcomes of CAT vs. FAT.

It was highlighted by a single study [23] that CAT was associated with less discomfort compared to FAT with a significant difference during the first week of treatment. Secondary to this, the authors in [23] also found that the patients in the CAT group consumed less pain medication than the patients in the FAT group. Even though the consumption of pain medication consistently was greater in the FAT group, the only significant difference was on the second day of treatment. When experiencing discomfort or pain during CAT, the patient can remove the appliance (for a short time), while patients undergoing FAT cannot remove the appliance. The absence of pain and/or discomfort has been emphasized by another study [36]; comfort and quality of life were deemed some of the most important factors when considering what type of orthodontic treatment to choose by prospective orthodontic patients. Regarding the consumption of analgesics, the results are comparable with another study [37], where a higher consumption of pain medication also was noted in the FAT group compared to the CAT (Invisalign^®^) group, although this was not significantly higher. In conflict with the findings of this review, Alajmi et al. (2020) [37] found that the mean value of pain level was almost identical in both groups.

One study [21] found that CAT was related to positive changes in body posture compared to the control group, who did not receive any treatment during the study period. It has previously been noted in the literature [38] that there is a relationship between jaw position and body posture, yet only in the upper part of the spine.

### 4.4. Disadvantages of CAT

Higher scores in the PSQI and on the ESS were reported in CAT compared to FAT, although no significant differences were found [24]. The same tendency was seen in other studies [23,37]; however, no significant differences were found.

Changes in speech have been registered as being more distinct in CAT when compared to FAT [25,26]. These results are supported by the literature [37], although clear aligners are not the only type of orthodontic appliance that might affect speech [39].

A single study [12] highlighted that FAT is considered to be better at achieving occlusal contacts and achieving all orthodontic movements when compared to CAT. In particular, root movements and extrusions are considered difficult to achieve with clear aligners, and refinements are often needed [12]. These viewpoints are supported by the literature [1,30,40] in terms of why CAT is still, in most cases, considered to be inferior to FAT.

### 4.5. Adverse Effects of CAT

Microplastics <5 µm that are detached from clear aligners due to mechanical friction are able to cross membranes. However, the group of microplastics <5 µm in the in vitro study [28] is so small that the use of clear aligners in a short period of time is considered safe by the authors. At present, there are no studies investigating the presence of microplastics in orthodontic patients being treated with clear aligners. However, this could be of great importance with patient safety in mind since micro- and nanoplastics pose a potential health risk [41].

The volume of the condyle bone was found to be decreasing in patients receiving CAT compared to patients receiving FAT [29]. In the literature, a single study [42] highlighted that CAT might have an impact on TMDs during treatment.

### 4.6. Limitations

An exclusion criterion for this review was studies investigating the primary or mixed dentition. Even though studies only investigating the permanent dentition were included, the age span in some of the studies included adolescents who might differ in their behavior regarding oral health and hygiene [43].

The only study included to highlight indications and contraindications in CAT is a cross-sectional study among orthodontists [12]. Cross-sectional studies are subject to nonresponse bias since the opinions of the respondents included cannot be held against the opinions of the orthodontists who did not respond to the questionnaire, which makes it hard to conclude whether the results are representative [44]. Furthermore, the response rate for this study was 3.8%, which is to be considered below the optimal goal [45]. None of the other studies included were investigating potential indications or contraindications for CAT, which makes it difficult to state any clear results regarding indications and contraindications for CAT.

Different methods for assessing the outcome of interest such as OIEARR, oral health, and oral hygiene were used.

Of the 52 studies screened by full-text reading, 18 of these were excluded due to the lack of control group. The evidence in CAT is still considered insufficient [1,30], which stresses the need for more high-quality RCTs.

Another limitation of this systematic review is the observation of heterogeneity between the included studies. It should be noted that both clinical and methodological heterogeneity [46] is present; however, this is likely to be considered quite inevitable when taking the various outcomes of interest in this study into account.

## 5. Conclusions

Based on the studies included, the following conclusions were made:

- Indications for CAT are mild to moderate malocclusions.
- Contraindications for CAT are severe malocclusions with impactions or severe craniofacial skeletal deviations.
- CAT is still considered inferior to FAT in more complex cases.
- Multiple advantages are present in CAT, but multiple disadvantages are also present in CAT that clinicians should take into account.
- Few adverse effects in CAT were presented, such as microplastics that might pose a potential health risk and the reduction in mandibular condyle bone after CAT.
- In order to draw a more accurate conclusion regarding the indications and contraindications in CAT, more high-quality studies are needed in future research.

## Figures and Tables

**Table 1:** Literature search terms for the PubMed database.

Literature search terms (PubMed)	(“Orthodontics”[MeSH Terms] OR “Malocclusion”[MeSH Terms] OR “orthodontic appliances, removable”[MeSH Terms] OR (“Orthodontics”[Text Word] OR “teeth straightening”[Text Word] OR “Malocclusion”[Text Word] OR “orthodontic appliance”[Text Word] OR “removable orthodontic appliance”[Text Word])) AND (“clear aligner*”[Text Word] OR “clear aligner treatment”[Text Word] OR “CAT”[Text Word] OR “invisalign”[Text Word] OR “aligner*”[Text Word]) AND (“indication*”[Text Word] OR “advantage*”[Text Word] OR “disadvantage*”[Text Word] OR “contraindication*”[Text Word] OR “adverse effects”[Text Word] OR “adverse effect”[Text Word] OR “pitfall*”[Text Word])

**Table 2:** Summary of characteristics of included studies regarding indications for CAT.

Author	Study Design	Study Objectives	Indication
Abu-Arqub et al. (2023) [12]	Cross-sectional	Investigate CAT implications and preferences among orthodontists in Canada and USA	Mild to moderate malocclusions

**Table 3:** Summary of characteristics of included studies regarding contraindications in CAT.

Author	Study Design	Study Objectives	Contraindication
Abu-Arqub et al. (2023) [12]	Cross-sectional	Investigate CAT implications and preferences among orthodontists in Canada and USA	Complex malocclusions with impactions or severe skeletal problems

**Table 4:** Summary of characteristics of included studies regarding advantages in CAT.

Author	Study Design	Study Objectives	Advantage
Kim et al. (2024) [13]	Quasi-experimental	Compare and analyze overall health status, oral hygiene management, and satisfaction CAT vs. FAT	Better gingival status compared to FAT Lower levels of oral cocci over time compared to FAT
Kurnaz et al. (2024) [14]	Retrospective	Investigate and compare external apical root resorption in patients undergoing CAT or FAT	Prevalence and severity of external apical root resorption is lower compared to FAT
Liou et al. (2024) [15]	Retrospective	Objective comparison of patient outcomes between CAT and FAT after “surgery-first” orthognathic surgery	Higher extent of improvement in the Peer Assessment Rating (PAR) index at T1 compared to FAT
de Leyva et al. (2023) [16]	RCT	Compare periodontal health and quality of life (QoL) between CAT and FAT (orthognathic surgery, surgery first)	Better periodontal health and QoL in CAT compared to FAT
Liao et al. (2021) [17]	Prospective	Characteristics and dental indices of patients CAT vs. FAT	CAT was found to be more esthetically pleasing compared to FAT
Gao et al. (2021) [18]	Prospective	Compare pain perception, anxiety, and OHRQoL CAT vs. FAT	Lower pain levels, less anxiety, and higher OHRQoL in CAT compared to FAT
Mummolo et al. (2019) [19]	Observational	Do removable appliances (clear aligners in particular) reduce the concentrations of s. mutans and lactobacilli in saliva compared to fixed appliances?	Lower risk for developing caries in CAT compared to FAT (10% vs. 40% in high risk of developing caries)
Eissa et al. (2018) [20]	Pilot	Evaluate the degree of orthodontically induced apical root resorption following treatment with Smart-Track aligners or two different fixed appliances	Lower degree of orthodontically induced apical root resorption in the aligner group compared to the fixed appliance group (conventional) in cases of class I malocclusion with mild to moderate crowding
Parrini et al. (2018) [21]	Controlled clinical trial	Investigate if Invisalign^®^ treatment produces changes to the posture during treatment	Better results in terms of positive correspondence between body posture, spine position, and occlusal contacts in the Invisalign^®^ group compared to the control group
Chhibber et al. (2017) [22]	RCT	Evaluate the oral hygiene in patients treated with clear aligners, self-ligated brackets, and elastomeric-ligated brackets	In the short term, better gingival index and papillary bleeding index for CAT compared to fat (but no significant difference in oral hygiene between the three groups)
White et al. (2017) [23]	RCT	Compare discomfort levels CAT (Invisalign^®^) vs. FAT. Secondary comparison of effect on pain medication and sleep disturbances	Less discomfort in CAT vs. FAT. Fewer patients took pain medication in the CAT group vs. the FAT group (but only one day where the difference was significant)

**Table 5:** Summary of characteristics of included studies regarding disadvantages in CAT.

Author	Study Design	Study Objectives	Disadvantage
Kurnaz et al. (2024) [14]	Retrospective	Investigate and compare external apical root resorption in patients undergoing CAT or FAT	External apical root resorption present in CAT
Hakami et al. (2023) [24]	Cross-sectional	Compare the quality of sleep between patients undergoing CAT or FAT	Higher PSQI and ESS score for CAT vs. FAT (but not significant)
Wang et al. (2023) [25]	Repeated-measure experimental study	Investigate and compare immediate soft tissue changes and asses the self-reported impact on speech by clear aligners and fixed appliances	Clear aligners have a significantly greater impact on speech than fixed appliances
Abu-Arqub et al. (2023) [12]	Cross-sectional	Investigate CAT implications and preferences among orthodontists in Canada and USA	The majority is of the opinion that FAT has better 3D control for all movements and results in better occlusal contacts compared to CAT. Refinements are often required to reach desired goals in CAT. Challenging to perform: ‑ Root movements; ‑ Extrusion.
Damasceno Melo et al. (2021) [26]	RCT	Evaluate speech production in patients undergoing CAT vs. FAT	Changes in speech in the CAT group identified by a speech therapist in the beginning of treatment (but adaptation was seen during treatment + self-perception of speech production was changed regardless of orthodontic appliance)
Eissa et al. (2018) [20]	Pilot	Evaluate the degree of orthodontically induced apical root resorption following treatment with Smart-Track aligners or two different fixed appliances	Orthodontically induced apical root resorption
Iglesias-Linares et al. (2017) [27]	Case–control	Determine whether CAT is associated with orthodontically induced external apical root resorption	Similar predisposition to experience OIEARR in Invisalign^®^ as in FAT

**Table 6:** Summary of characteristics of included studies regarding adverse effects in CAT.

Author	Study Design	Study Objectives	Adverse Effect
Quinzi et al. (2023) [28]	In vitro	Investigate possible detachment of microplastics from clear aligners (compares different aligner manufacturers)	Microparticles smaller than 5 µm are able to cross membranes (but this group was very small)
Ertugrul et al. (2022) [29]	Retrospective	Examine the effect of CAT and FAT on mandibular condyle bone density and formation	Reduced size of the condyle (increased in FAT)

## Data Availability

No new data were created or analyzed in this study. Data sharing is not applicable to this article.
